# Anomalous ion diffusion within skeletal muscle transverse tubule networks

**DOI:** 10.1186/1742-4682-4-18

**Published:** 2007-05-17

**Authors:** Paul R Shorten, Tanya K Soboleva

**Affiliations:** 1 AgResearch Limited Ruakura Research Centre, Private Bag 3123, Hamilton, New Zealand

## Abstract

**Background:**

Skeletal muscle fibres contain transverse tubular (t-tubule) networks that allow electrical signals to rapidly propagate into the fibre. These electrical signals are generated by the transport of ions across the t-tubule membranes and this can result in significant changes in ion concentrations within the t-tubules during muscle excitation. During periods of repeated high-frequency activation of skeletal muscle the t-tubule K^+ ^concentration is believed to increase significantly and diffusive K^+ ^transport from the t-tubules into the interstitial space provides a mechanism for alleviating muscle membrane depolarization. However, the tortuous nature of the highly branched space-filling t-tubule network impedes the diffusion of material through the network. The effective diffusion coefficient for ions in the t-tubules has been measured to be approximately five times lower than in free solution, which is significantly different from existing theoretical values of the effective diffusion coefficient that range from 2–3 times lower than in free solution. To resolve this discrepancy, in this paper we study the process of diffusion within electron microscope scanned sections of the skeletal muscle t-tubule network using mathematical modelling and computer simulation techniques. Our model includes t-tubule geometry, tautness, hydrodynamic and non-planar network factors.

**Results:**

Using our model we found that the t-tubule network geometry reduced the K^+ ^diffusion coefficient to 19–27% of its value in free solution, which is consistent with the experimentally observed value of 21% and is significantly smaller than existing theoretical values that range from 32–50%. We also found that diffusion in the t-tubules is anomalous for skeletal muscle fibres with a diameter of less than approximately 10–20 μm as a result of obstructed diffusion. We also observed that the [K^+^] within the interior of the t-tubule network during high-frequency activation is greater for fibres with a larger diameter. Smaller skeletal muscle fibres are therefore more resistant to membrane depolarization. Because the t-tubule network is anisotropic and inhomogeneous, we also found that the [K^+^] distribution generated within the network was irregular for fibres of small diameter.

**Conclusion:**

Our model explains the measured effective diffusion coefficient for ions in skeletal muscle t-tubules.

## 1. Background

Skeletal muscle fibres contain transverse tubular (t-tubule) networks that provide for rapid propagation of electrical signals into the fibre and ensures near simultaneous contraction in the constituent myofibrils. These t-tubule networks are highly branched space-filling networks that are located near sarcomere Z-lines in amphibians and the A-I junction in mammals. The t-tubule network therefore largely lies in a plane perpendicular to the axis of the muscle fibre. The electrical signals that propagate along the planar t-tubules are generated by the transport of ions across the t-tubule membranes and this can result in significant changes in ion concentrations within the t-tubules during muscle excitation. In particular, K^+ ^ions accumulate within the t-tubule network as a result of voltage-gated K^+ ^channels that repolarize the membrane during action potentials. This K^+ ^accumulation can result in membrane depolarization and reduced membrane excitability as a result of Na^+ ^channel inactivation and consequently reduced muscle force output [1]. The K^+ ^ionic gradients across the t-tubule membranes are re-established by the Na^+^-K^+ ^exchanger following an action potential [2]. During periods of repeated high-frequency activation of skeletal muscle the t-tubule K^+ ^concentration ([K^+^]_*t*_) is believed to increase significantly [3,4] and diffusive K^+ ^transport from the t-tubules into the interstitial space provides a further mechanism for alleviating muscle membrane depolarization. In this paper we study the process of K^+ ^diffusion within the skeletal muscle t-tubule network using mathematical modelling and computer simulation techniques.

The structure of biological tissue impedes the diffusion and transport of material through the tissue. In particular it has been calculated that the effective diffusion coefficient for ions in the t-tubules is approximately five times lower than in free solution [5,6]. In this paper we analyze diffusion on the skeletal muscle t-tubule networks imaged by Peachey and Eisenberg [7] and Hayashi et al. [8]. We have found that the t-tubule network restricts the diffusion of material through the network as a result of geometric, hydrodynamic and network tautness factors. We also find that diffusion in the t-tubules over short distances is anomalous (i.e. does not obey Brownian motion).

## 2. Results

### Diffusion within the t-tubule network

**Figure 1:**
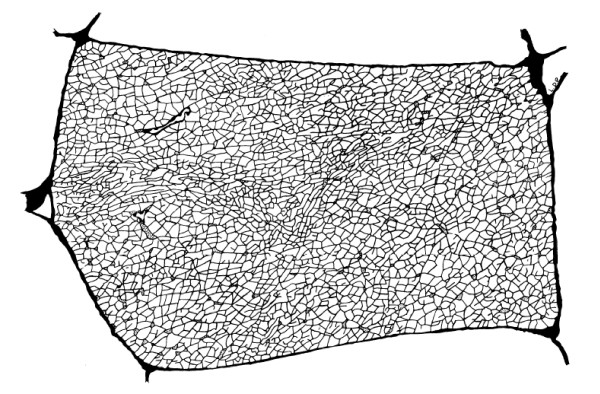
A reconstruction of the t-tubule network made by Peachey and Eisenberg [7] using electron microscope slices of frog sartorius muscle fibres (×1400; fibre is approximately 40 × 80 μm). Reprinted with permission from the Biophysical Society and L. D. Peachey.

Electron micrographs suggest that t-tubule cross sections are very near to circular [9]. The diameter of the t-tubules is approximately 18 nm [10] and the diameter of skeletal muscle fibres range from 5–100 μm. T-tubule branch lengths are approximately 1 μm, which is roughly the diameter of the myofibrils [11,12]. Images of the t-tubule system have been presented by a number of different authors [7,9,12]. A reconstruction of the t-tubule network by Peachey and Eisenberg [7] using electron microscope slices of frog sartorius muscle fibres is shown in Fig. 1. Dead end tubules are quite common and although there are a few regions where the tubules are preferentially directed, the network is largely isotropic and irregular. T-tubule branch intersections are termed nodes and the average number of t-tubule branches per node is about 3.2 [13]. The t-tubule network parameter statistics have been calculated in the guinea pig, frog and human skeletal muscle fibres [8,11-13].

The microstructure of biological tissue limits the flow of solutes through it in a manner dependent on the geometry of the tissue. The resistive properties of tissue are described by tortuosity factors, which have been calculated for a number of different tissue types and different fixed regular geometries [13,14]. The tortuosity factor, *τ*, is defined by the following homogenised diffusion equation [15,16]:

∂c∂t=τD∇2c=Dapp∇2c
 MathType@MTEF@5@5@+=feaafiart1ev1aaatCvAUfKttLearuWrP9MDH5MBPbIqV92AaeXatLxBI9gBaebbnrfifHhDYfgasaacH8akY=wiFfYdH8Gipec8Eeeu0xXdbba9frFj0=OqFfea0dXdd9vqai=hGuQ8kuc9pgc9s8qqaq=dirpe0xb9q8qiLsFr0=vr0=vr0dc8meaabaqaciaacaGaaeqabaqabeGadaaakeaadaWcaaqaaiabgkGi2kabdogaJbqaaiabgkGi2kabdsha0baacqGH9aqpiiGacqWFepaDcqWGebarcqGHhis0daahaaWcbeqaaiabikdaYaaakiabdogaJjabg2da9iabdseaenaaCaaaleqabaGaemyyaeMaemiCaaNaemiCaahaaOGaey4bIe9aaWbaaSqabeaacqaIYaGmaaGccqWGJbWyaaa@4492@

where *D *is the temperature-dependent solute diffusion coefficient in free solution, *c *is the solute concentration in the tissue, *t *is time and *D*^*app *^is the apparent diffusion coefficient. The tortuosity factor then determines the difference between the apparent diffusion coefficient in the tissue and the diffusion coefficient in free solution. This equation then describes the macroscopic transport of the solute through the tissue. The mean squared displacement (MSD) of the solute is

⟨*r*^2^(*t*)⟩ = 2*dτDt*

where *d *is the spatial dimensionality. Several regular two-dimensional networks of one-dimensional t-tubules have been used to model the t-tubule geometry. Tortuosity factors have been calculated for these networks using flux density analysis and homogenization methods and it has been found that *τ *= 0.5 for regular hexagonal, square and trigonal network geometries and *τ *= 0.4 for staggered square network geometries [13,17-19]. Furthermore, using theoretical methods, Mathias et al. [13] have estimated that *τ *= 0.32 from the morphometric parameters of frog skeletal muscle t-tubule networks. The irregular structure of the t-tubule network therefore significantly reduces diffusion on the network.

Experimental measurements of the t-tubule tortuosity factor have been made for a number of different ionic species. Tortuosity measurements range from *τ *= 0.11 for Ca^2+ ^to *τ *= 0.21 for K^+^, which are significantly lower than the tortuosity factors calculated on theoretical grounds (0.32 ≤ *τ *≤ 0.5). For example, Fujishiro and Kawata [6] investigated Na^+^ diffusion into the t-tubule network when frog skeletal muscle fibres were moved from a Na^+  ^free solution into normal Ringer solution with [Na^+^] = 111 mM. The authors employed a diffusion equation and found that the diffusion of Na^+ ^along the transverse tubules was consistent with an apparent diffusion coefficient of DNaapp
 MathType@MTEF@5@5@+=feaafiart1ev1aaatCvAUfKttLearuWrP9MDH5MBPbIqV92AaeXatLxBI9gBaebbnrfifHhDYfgasaacH8akY=wiFfYdH8Gipec8Eeeu0xXdbba9frFj0=OqFfea0dXdd9vqai=hGuQ8kuc9pgc9s8qqaq=dirpe0xb9q8qiLsFr0=vr0=vr0dc8meaabaqaciaacaGaaeqabaqabeGadaaakeaacqWGebardaqhaaWcbaGaemOta4KaemyyaegabaGaemyyaeMaemiCaaNaemiCaahaaaaa@3477@ = 3.11 × 10^-6 ^cm^2^/s, which is significantly less than that for an aqueous solution at 25°C (14.8 × 10^-6 ^cm^2^/s). Using Markov chain Monte Carlo we found that the SEM associated with this calculated apparent diffusion coefficient is 0.3 cm^2^/s. This data is consistent with a t-tubule network total tortuosity factor of *τ *= 0.21 ± 0.02. Almers [5] investigated K^+ ^diffusion in frog sartorius t-tubules using a voltage-clamp technique at 22°C. Using a computer model they found that their data was consistent with an apparent t-tubule K^+ ^diffusion coefficient of DKapp
 MathType@MTEF@5@5@+=feaafiart1ev1aaatCvAUfKttLearuWrP9MDH5MBPbIqV92AaeXatLxBI9gBaebbnrfifHhDYfgasaacH8akY=wiFfYdH8Gipec8Eeeu0xXdbba9frFj0=OqFfea0dXdd9vqai=hGuQ8kuc9pgc9s8qqaq=dirpe0xb9q8qiLsFr0=vr0=vr0dc8meaabaqaciaacaGaaeqabaqabeGadaaakeaacqWGebardaqhaaWcbaGaem4saSeabaGaemyyaeMaemiCaaNaemiCaahaaaaa@3326@ = 3.8 × 10^-6 ^cm^2^/s. We found using Markov chain Monte Carlo that the SEM associated with this calculated apparent diffusion coefficient is 0.1 cm^2^/s. The K^+ ^diffusion coefficient in free solution at 25°C is 18.3 × 10^-6 ^cm^2^/s [20]. This data is consistent with a t-tubule network total tortuosity factor of *τ *= 0.21 ± 0.005. Almers et al. [21] investigated Ca^2+ ^depletion in frog muscle tubules using a voltage-clamp technique at 20–24°C. They found that their data was consistent with an apparent t-tubule Ca^2+ ^diffusion coefficient of DCaapp
 MathType@MTEF@5@5@+=feaafiart1ev1aaatCvAUfKttLearuWrP9MDH5MBPbIqV92AaeXatLxBI9gBaebbnrfifHhDYfgasaacH8akY=wiFfYdH8Gipec8Eeeu0xXdbba9frFj0=OqFfea0dXdd9vqai=hGuQ8kuc9pgc9s8qqaq=dirpe0xb9q8qiLsFr0=vr0=vr0dc8meaabaqaciaacaGaaeqabaqabeGadaaakeaacqWGebardaqhaaWcbaGaem4qamKaemyyaegabaGaemyyaeMaemiCaaNaemiCaahaaaaa@3461@ = 0.87 × 10^-6 ^cm^2^/s with a SEM of 0.18 cm^2^/s. The Ca^2+ ^diffusion coefficient in free solution at 25°C is 7.7 × 10^-6 ^cm^2^/s [22]. This data is therefore consistent with a t-tubule network total tortuosity factor of *τ *= 0.11 ± 0.02. Ca^2+ ^binding may possibly explain the lower tortuosity factor for Ca^2+^. The experimentally observed t-tubule network total tortuosity factors (0.11 ≤ *τ *≤ 0.21) are therefore significantly smaller than existing estimates of the tortuosity factor based on theoretical considerations (0.32 ≤ *τ *≤ 0.5).

### Anomalous and obstructed K^+ ^diffusion in the t-tubule network

The t-tubule network obstructs the diffusion of K^+ ^and this is referred to as obstructed diffusion [23]. A Monte Carlo random walk simulation technique to investigate obstructed diffusion has been proposed by Saxton [24,25] and Olveczky and Verkman, [26]. In this technique, tracers move by random walk throughout a mesh that defines an obstacle-free domain. Tracers are obstructed by the obstacles and a particle to be moved across an obstacle remains in its original position representing particle reflection at the obstacle boundary. If the obstacles in a two dimensional domain are defined by Ω^
 MathType@MTEF@5@5@+=feaafiart1ev1aaatCvAUfKttLearuWrP9MDH5MBPbIqV92AaeXatLxBI9gBaebbnrfifHhDYfgasaacH8akY=wiFfYdH8Gipec8Eeeu0xXdbba9frFj0=OqFfea0dXdd9vqai=hGuQ8kuc9pgc9s8qqaq=dirpe0xb9q8qiLsFr0=vr0=vr0dc8meaabaqaciaacaGaaeqabaqabeGadaaakeaaiiqacuWFPoWvgaqcaaaa@2E4F@, then a random walk **x**(t) on this domain is given by

x˜j+1=xj+ηj4DΔt,tj+1=tj+Δt,xj+1(tj+1)={x˜j+1,ifx˜j+1∉Ω^xj,ifx˜j+1∈Ω^
 MathType@MTEF@5@5@+=feaafiart1ev1aaatCvAUfKttLearuWrP9MDH5MBPbIqV92AaeXatLxBI9gBaebbnrfifHhDYfgasaacH8akY=wiFfYdH8Gipec8Eeeu0xXdbba9frFj0=OqFfea0dXdd9vqai=hGuQ8kuc9pgc9s8qqaq=dirpe0xb9q8qiLsFr0=vr0=vr0dc8meaabaqaciaacaGaaeqabaqabeGadaaakqaabeqaaiqbhIha4zaaiaWaaSbaaSqaaiabdQgaQjabgUcaRiabigdaXaqabaGccqGH9aqpcqWH4baEdaWgaaWcbaGaemOAaOgabeaakiabgUcaRGGadiab=D7aOnaaBaaaleaacqWGQbGAaeqaaOWaaOaaaeaacqaI0aancqWGebarcqqHuoarcqWG0baDaSqabaGccqGGSaalaeaacqWG0baDdaWgaaWcbaGaemOAaOMaey4kaSIaeGymaedabeaakiabg2da9iabdsha0naaBaaaleaacqWGQbGAaeqaaOGaey4kaSIaeuiLdqKaemiDaqNaeiilaWcabaGaeCiEaG3aaSbaaSqaaiabdQgaQjabgUcaRiabigdaXaqabaGcdaqadaqaaiabdsha0naaBaaaleaacqWGQbGAcqGHRaWkcqaIXaqmaeqaaaGccaGLOaGaayzkaaGaeyypa0ZaaiqaaeaafaqabeGadaaabaGafCiEaGNbaGaadaWgaaWcbaGaemOAaOMaey4kaSIaeGymaedabeaakiabcYcaSaqaaGqaaiab+LgaPjab+zgaMbqaaiqbhIha4zaaiaWaaSbaaSqaaiabdQgaQjabgUcaRiabigdaXaqabaGccqGHjiYZcuqHPoWvgaqcaaqaaiabhIha4naaBaaaleaacqWGQbGAaeqaaOGaeiilaWcabaGae4xAaKMae4NzaygabaGafCiEaGNbaGaadaWgaaWcbaGaemOAaOMaey4kaSIaeGymaedabeaakiabgIGiolqbfM6axzaajaaaaaGaay5Eaaaaaaa@79F2@

where Δ*x *= 4DΔt
 MathType@MTEF@5@5@+=feaafiart1ev1aaatCvAUfKttLearuWrP9MDH5MBPbIqV92AaeXatLxBI9gBaebbnrfifHhDYfgasaacH8akY=wiFfYdH8Gipec8Eeeu0xXdbba9frFj0=OqFfea0dXdd9vqai=hGuQ8kuc9pgc9s8qqaq=dirpe0xb9q8qiLsFr0=vr0=vr0dc8meaabaqaciaacaGaaeqabaqabeGadaaakeaadaGcaaqaaiabisda0iabdseaejabfs5aejabdsha0bWcbeaaaaa@31A5@ is the mesh size, Δ*t *is the time step size and **η**_*j *_is a random unit vector in one of the four Cartesian axis directions. An ensemble of tracer paths can then be used to calculate the mean squared displacement (⟨*r*^2^(*t*)⟩) and the effective diffusion coefficient respectively [24]

**Figure 2:**
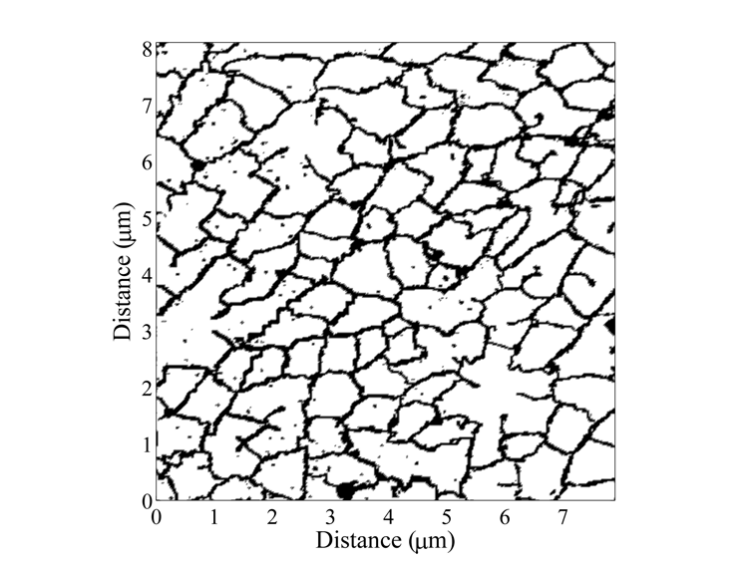
A scanned section of the human skeletal muscle t-tubule network from Hayashi et al. [8] (×8000; the scanned section is approximately 8 × 8 μm).

〈ri2(t)〉=〈‖xi(t)−xi0‖22〉,τi=DiappD=lim⁡t→∞〈ri2(t)〉2Dt,
 MathType@MTEF@5@5@+=feaafiart1ev1aaatCvAUfKttLearuWrP9MDH5MBPbIqV92AaeXatLxBI9gBaebbnrfifHhDYfgasaacH8akY=wiFfYdH8Gipec8Eeeu0xXdbba9frFj0=OqFfea0dXdd9vqai=hGuQ8kuc9pgc9s8qqaq=dirpe0xb9q8qiLsFr0=vr0=vr0dc8meaabaqaciaacaGaaeqabaqabeGadaaakqaabeqaamaaamaabaGaemOCai3aa0baaSqaaiabdMgaPbqaaiabikdaYaaakmaabmaabaGaemiDaqhacaGLOaGaayzkaaaacaGLPmIaayPkJaGaeyypa0ZaaaWaaeaadaqbdaqaaiabdIha4naaBaaaleaacqWGPbqAaeqaaOWaaeWaaeaacqWG0baDaiaawIcacaGLPaaacqGHsislcqWG4baEdaWgaaWcbaGaemyAaKMaeGimaadabeaaaOGaayzcSlaawQa7amaaDaaaleaacqaIYaGmaeaacqaIYaGmaaaakiaawMYicaGLQmcacqGGSaalaeaaiiGacqWFepaDdaWgaaWcbaGaemyAaKgabeaakiabg2da9maalaaabaGaemiraq0aa0baaSqaaiabdMgaPbqaaiabdggaHjabdchaWjabdchaWbaaaOqaaiabdseaebaacqGH9aqpdaWfqaqaaiGbcYgaSjabcMgaPjabc2gaTbWcbaGaemiDaqNaeyOKH4QaeyOhIukabeaakmaalaaabaWaaaWaaeaacqWGYbGCdaqhaaWcbaGaemyAaKgabaGaeGOmaidaaOWaaeWaaeaacqWG0baDaiaawIcacaGLPaaaaiaawMYicaGLQmcaaeaacqaIYaGmcqWGebarcqWG0baDaaGaeiilaWcaaaa@6CE8@

where **x**(*t*) = [*x*_1_(*t*) *x*_2_(*t*)] and **x**_0 _= [*x*_10 _*x*_20_]. We applied this technique to calculate random walks on scanned skeletal muscle t-tubule network geometries from Peachey and Eisenberg [7] and Hayashi et al. [8] (Fig. 2) with Δ*x *= 100 nm and 20 nm respectively. Tracer 1.71 was used to scan the images.

**Figure 3:**
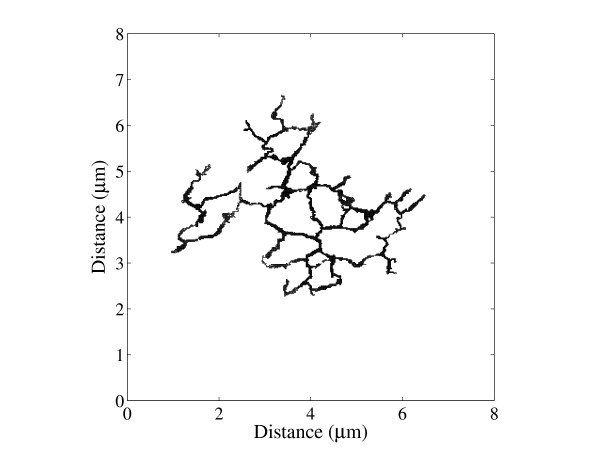
Simulated random walk of a single K^+ ^ion within the human skeletal muscle t-tubule network (starting from the centre of the fibre).

**Figure 4:**
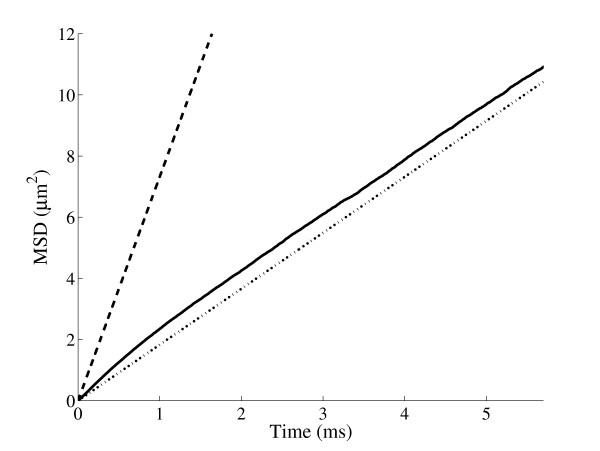
The mean squared displacement (MSD) in K^+ ^transport as a function of time for a random walk on the human t-tubule network (—) and an unrestricted random walk in two dimensions (– – –). The tubules significantly obstruct the transport of K^+ ^and the diffusion of K^+ ^in the t-tubule network is anomalous over short distances (i.e. the relationship between MSD and time is not linear as per standard Brownian diffusion (---)).

An example of a random walk on the human skeletal muscle network is shown in Fig. 3. A plot of the mean squared displacement (MSD) as a function of time is shown in Fig. 4 for a random walk on the t-tubule network and an unrestricted random walk in two dimensions. The MSD is lower in the t-tubules and diffusion in the t-tubules is anomalous over short distances, i.e.

⟨*r*^2^(*t*)⟩ = *K_α_ t*^*α*^,     *α *≠ 1

where *K_α _*is a generalised transport coefficient and *α *is the anomalous exponent, which is a measure of the irregular movement of the particle. The anomalous diffusion process described by Eq. (5) is referred to as subdiffusion if *α *< 1 and superdiffusion if *α *> 1. We found that *α *= 0.86, *K_α_*= 2.44 μm^2^ms^-*α *^for the human t-tubule geometry and *α *= 0.91, *K_α_*= 3.16 μm^2^ms^-*α *^for the frog t-tubule geometry. In both cases *α *is greater than the percolation threshold 0.697 [27] and therefore the obstructed diffusion is a localised phenomenon. Diffusion is therefore anomalous for short distances whereas over large distances diffusion is normal. The crossover length (R*) between anomalous and normal diffusion was estimated according to the definition [24]

〈r2(t)〉~{tαt, r<R∗r>R∗.
 MathType@MTEF@5@5@+=feaafiart1ev1aaatCvAUfKttLearuWrP9MDH5MBPbIqV92AaeXatLxBI9gBaebbnrfifHhDYfgasaacH8akY=wiFfYdH8Gipec8Eeeu0xXdbba9frFj0=OqFfea0dXdd9vqai=hGuQ8kuc9pgc9s8qqaq=dirpe0xb9q8qiLsFr0=vr0=vr0dc8meaabaqaciaacaGaaeqabaqabeGadaaakeaadaaadeqaaiabdkhaYnaaCaaaleqabaGaeGOmaidaaOGaeiikaGIaemiDaqNaeiykaKcacaGLPmIaayPkJaGaeiOFa43aaiqabeaafaqaaeGabaaabaGaemiDaq3aaWbaaSqabeaaiiGacqWFXoqyaaaakeaacqWG0baDaaGaeiilaWIaeeiiaasbaeqabiqaaaqaaGqaciab+jhaYHGaaiab9Xda8iab+jfasnaaCaaaleqabaGaey4fIOcaaaGcbaGaemOCaiNaeyOpa4JaemOuai1aaWbaaSqabeaacqGHxiIkaaaaaaGccaGL7baacqGGUaGlaaa@47C6@

We found that R* = 5.5 μm for the human t-tubule geometry and R* = 7.5 μm for the frog t-tubule geometry. It follows that K^+ ^diffusion in the t-tubule network is anomalous for skeletal muscle fibres with a diameter of less than approximately 11–15 μm (i.e. small skeletal muscle fibres). For these muscle fibres subdiffusion processes are important, and consequently Eq. (1) is not valid. Diffusion can be described by the fractional diffusion equation [23]

∂c∂t=Dt     1−α0Kα∂2c∂r2,

where the Riemann-Liouville operator Dt      1−α0, for 0 <*α *< 1, is defined by

Dt      1−α0x(t)=1Γ(α)∂∂t∫0tx(s)(t−s)1−αds,

where Γ(*α*) is the Gamma function.

On sections of the t-tubule network with a diameter greater than approximately 11–15 μm diffusion can be described by the standard homogenised diffusion equation (Eq. 1). The random walk simulations can therefore be used to calculate the tissue tortuosity factors. T-tubule network tortuosity factors of *τ *= 0.25 and *τ *= 0.35 were calculated for the human and frog skeletal muscle t-tubule geometries respectively. The geometric tortuosity factors for these networks calculated by random walk simulation are consistent with the previous findings of Mathias et al. [13] who used flux density analysis to estimate that *τ *= 0.32 from the morphometric parameters of frog skeletal muscle t-tubule networks. These theoretical estimates of the t-tubule network tortuosity factor (*τ *= 0.25, 0.35, 0.32) are nonetheless significantly larger than experimentally observed total tortuosity factors (0.11 ≤ *τ *≤ 0.21). We believe that this inconsistency is due to non-planar and hydrodynamic effects. If we include these effects into our theoretical model then we obtain a tortuosity factor that is consistent with the experimental data.

Skeletal muscle t-tubule networks are generally conceptualised as planar networks perpendicular to the fibre direction that are located at the boundary between the A-band and I-band regions in vertebrate fibres. Nevertheless, electron microscope measurements of longitudinal cross-sections of human skeletal muscle t-tubule networks indicate that the network is not planar and lies within a region about 300–400 nm in width [8]. T-tubule branches are therefore greater in length than they appear in two-dimensional transverse images of t-tubule networks. We estimate from images of longitudinal cross-sections of t-tubule networks by Hayashi et al. [8] that the t-tubule branch lengths are approximately 10% greater than depicted in Fig. 2. From the relationship between the diffusion coefficient, space and time defined by Eq. (2), it follows that that the effective diffusion coefficient on this non-planar t-tubule network is reduced by the factor 1/1.1^2 ^= 0.83.

Friedrich et al. [16] identified that because of the small diameter of the t-tubules hydrodynamic wall effects reduce the diffusion of Ca^2+ ^in the tubules (DCaTube
 MathType@MTEF@5@5@+=feaafiart1ev1aaatCvAUfKttLearuWrP9MDH5MBPbIqV92AaeXatLxBI9gBaebbnrfifHhDYfgasaacH8akY=wiFfYdH8Gipec8Eeeu0xXdbba9frFj0=OqFfea0dXdd9vqai=hGuQ8kuc9pgc9s8qqaq=dirpe0xb9q8qiLsFr0=vr0=vr0dc8meaabaqaciaacaGaaeqabaqabeGadaaakeaacqWGebardaqhaaWcbaGaem4qamKaemyyaegabaGaemivaqLaemyDauNaemOyaiMaemyzaugaaaaa@3588@) relative to that in free solution (DCasol
 MathType@MTEF@5@5@+=feaafiart1ev1aaatCvAUfKttLearuWrP9MDH5MBPbIqV92AaeXatLxBI9gBaebbnrfifHhDYfgasaacH8akY=wiFfYdH8Gipec8Eeeu0xXdbba9frFj0=OqFfea0dXdd9vqai=hGuQ8kuc9pgc9s8qqaq=dirpe0xb9q8qiLsFr0=vr0=vr0dc8meaabaqaciaacaGaaeqabaqabeGadaaakeaacqWGebardaqhaaWcbaGaem4qamKaemyyaegabaGaem4CamNaem4Ba8MaemiBaWgaaaaa@347B@). This phenomenon is called hindered diffusion and contributes to the observed t-tubule network total tortuosity factor. The reduction in diffusion is [28]

DCaTubeDCasol=[1+(9/8)λln⁡λ−1.539λ+1.2λ2]
 MathType@MTEF@5@5@+=feaafiart1ev1aaatCvAUfKttLearuWrP9MDH5MBPbIqV92AaeXatLxBI9gBaebbnrfifHhDYfgasaacH8akY=wiFfYdH8Gipec8Eeeu0xXdbba9frFj0=OqFfea0dXdd9vqai=hGuQ8kuc9pgc9s8qqaq=dirpe0xb9q8qiLsFr0=vr0=vr0dc8meaabaqaciaacaGaaeqabaqabeGadaaakeaadaWcaaqaaiabdseaenaaDaaaleaacqWGdbWqcqWGHbqyaeaacqWGubavcqWG1bqDcqWGIbGycqWGLbqzaaaakeaacqWGebardaqhaaWcbaGaem4qamKaemyyaegabaGaem4CamNaem4Ba8MaemiBaWgaaaaakiabg2da9maadmaabaGaeGymaeJaey4kaSIaeiikaGIaeGyoaKJaei4la8IaeGioaGJaeiykaKccciGae83UdWMagiiBaWMaeiOBa4Mae83UdWMaeyOeI0IaeGymaeJaeiOla4IaeGynauJaeG4mamJaeGyoaKJae83UdWMaey4kaSIaeGymaeJaeiOla4IaeGOmaiJae83UdW2aaWbaaSqabeaacqaIYaGmaaaakiaawUfacaGLDbaaaaa@5AE7@

where *λ *= *r*_*Ca*_/*r*_*Tube *_is the solute/t-tubule size ratio. Potassium has an atomic radius of 0.22 nm and the t-tubules are on average 18 nm in diameter so that the solute/t-tubule size ratio is

λ=rKrTube=0.2218=0.012
 MathType@MTEF@5@5@+=feaafiart1ev1aaatCvAUfKttLearuWrP9MDH5MBPbIqV92AaeXatLxBI9gBaebbnrfifHhDYfgasaacH8akY=wiFfYdH8Gipec8Eeeu0xXdbba9frFj0=OqFfea0dXdd9vqai=hGuQ8kuc9pgc9s8qqaq=dirpe0xb9q8qiLsFr0=vr0=vr0dc8meaabaqaciaacaGaaeqabaqabeGadaaakeaaiiGacqWF7oaBcqGH9aqpdaWcaaqaaiabdkhaYnaaBaaaleaacqWGlbWsaeqaaaGcbaGaemOCai3aaSbaaSqaaiabdsfaujabdwha1jabdkgaIjabdwgaLbqabaaaaOGaeyypa0ZaaSaaaeaacqaIWaamcqGGUaGlcqaIYaGmcqaIYaGmaeaacqaIXaqmcqaI4aaoaaGaeyypa0JaeGimaaJaeiOla4IaeGimaaJaeGymaeJaeGOmaidaaa@4588@

and DKTube/DKsol=0.92
 MathType@MTEF@5@5@+=feaafiart1ev1aaatCvAUfKttLearuWrP9MDH5MBPbIqV92AaeXatLxBI9gBaebbnrfifHhDYfgasaacH8akY=wiFfYdH8Gipec8Eeeu0xXdbba9frFj0=OqFfea0dXdd9vqai=hGuQ8kuc9pgc9s8qqaq=dirpe0xb9q8qiLsFr0=vr0=vr0dc8meaabaqaciaacaGaaeqabaqabeGadaaakeaadaWcgaqaaiabdseaenaaDaaaleaacqWGlbWsaeaacqWGubavcqWG1bqDcqWGIbGycqWGLbqzaaaakeaacqWGebardaqhaaWcbaGaem4saSeabaGaem4CamNaem4Ba8MaemiBaWgaaaaakiabg2da9iabicdaWiabc6caUiabiMda5iabikdaYaaa@3FD5@. K^+ ^diffusion in the t-tubules is therefore 8% slower than in free solution. Hydrodynamic wall effects within the small diameter t-tubules therefore have a small but significant effect on K^+ ^diffusion within the t-tubules.

Our modelling analysis therefore predicts a total tortuosity factor for K^+ ^within the t-tubule network of 0.30 × 0.92 × 0.83 = 0.23 (geometric, hydrodynamic and non-planar factors), which agrees well with the experimental observations of Almers et al. [5] who found a t-tubule network total tortuosity factor for K^+ ^of *τ *= 0.21. Although there is variability in the t-tubule branch diameters within the t-tubule network [10,29], and therefore variability in the level of hydrodynamic wall effects at different locations in the network, we found that this has a negligible effect on the t-tubule network total tortuosity factor for K^+^. We use a standard deviation in the diameter of the t-tubule branches of 1–3 nm [10,29] and assume that each t-tubule branch contains 3 segments of different diameter [29]. T-tubule network geometries are also different in different muscle fibres. For example, the t-tubule network from slow-twitch guinea pig skeletal muscle is more non-planar than that from fast-twitch skeletal muscle [12]. T-tubule branch lengths are 3% greater in length than in transverse images of t-tubules in fast-twitch guinea pig skeletal muscle and 30% greater in length than in transverse images of t-tubules in slow twitch fibres [12]. From the relationship between the diffusion coefficient, space and time defined by Eq. (2), it follows that the effective diffusion coefficient on a non-planar t-tubule network is reduced by the factor 1/1.03^2 ^= 0.94 in fast-twitch guinea pig skeletal muscle and by 1/1.3^2^= 0.59 in slow-twitch fibres. This suggests that the effective diffusion coefficient on the t-tubule network in slow-twitch fibres is 37% smaller than in fast-twitch fibres.

### The effect of the t-tubule network geometry on the [K^+^] distribution within t-tubules and membrane depolarization

During periods of repeated high-frequency activation of skeletal muscle the t-tubule K^+ ^concentration ([K^+^]_*t*_) is believed to increase significantly [3,4] as a result of voltage-gated K^+ ^channels that repolarize the membrane during action potentials. This K^+ ^accumulation results in membrane depolarization and reduced muscle force output [1]. The K^+ ^ionic gradients across the t-tubule membranes are re-established by diffusive K^+ ^transport from the t-tubules into the interstitial space and by K^+ ^transport across the t-tubule membranes by the Na^+^-K^+ ^exchanger [2]. A simple model of K^+ ^accumulation within the t-tubules during muscle activation is

∂c∂t=D(∂2c∂x2+∂2c∂y2),(x,y)∈Ωc(x,y,0)=c0c(x,y,t)=c0,(x,y)∈Γ,t>0−n^⋅∇c=B(A−c),(x,y)∈Ω′
 MathType@MTEF@5@5@+=feaafiart1ev1aaatCvAUfKttLearuWrP9MDH5MBPbIqV92AaeXatLxBI9gBaebbnrfifHhDYfgasaacH8akY=wiFfYdH8Gipec8Eeeu0xXdbba9frFj0=OqFfea0dXdd9vqai=hGuQ8kuc9pgc9s8qqaq=dirpe0xb9q8qiLsFr0=vr0=vr0dc8meaabaqaciaacaGaaeqabaqabeGadaaakqaabeqaauaabeqabiaaaeaadaWcaaqaaiabgkGi2kabdogaJbqaaiabgkGi2kabdsha0baacqGH9aqpcqWGebardaqadaqaamaalaaabaGaeyOaIy7aaWbaaSqabeaacqaIYaGmaaGccqWGJbWyaeaacqGHciITcqWG4baEdaahaaWcbeqaaiabikdaYaaaaaGccqGHRaWkdaWcaaqaaiabgkGi2oaaCaaaleqabaGaeGOmaidaaOGaem4yamgabaGaeyOaIyRaemyEaK3aaWbaaSqabeaacqaIYaGmaaaaaaGccaGLOaGaayzkaaGaeiilaWcabaWaaeWaaeaacqWG4baEcqGGSaalcqWG5bqEaiaawIcacaGLPaaacqGHiiIZcqqHPoWvaaaabaGaem4yam2aaeWaaeaacqWG4baEcqGGSaalcqWG5bqEcqGGSaalcqaIWaamaiaawIcacaGLPaaacqGH9aqpcqWGJbWydaWgaaWcbaGaeGimaadabeaaaOqaauaabeqabmaaaeaacqWGJbWydaqadaqaaiabdIha4jabcYcaSiabdMha5jabcYcaSiabdsha0bGaayjkaiaawMcaaiabg2da9iabdogaJnaaBaaaleaacqaIWaamaeqaaOGaeiilaWcabaWaaeWaaeaacqWG4baEcqGGSaalcqWG5bqEaiaawIcacaGLPaaacqGHiiIZcqqHtoWrcqGGSaalaeaacqWG0baDcqGH+aGpcqaIWaamaaaabaqbaeqabeGaaaqaaiabgkHiTGqabiqb=5gaUzaajaGaeyyXICTaey4bIeTaem4yamMaeyypa0JaemOqai0aaeWaaeaacqWGbbqqcqGHsislcqWGJbWyaiaawIcacaGLPaaacqGGSaalaeaadaqadaqaaiabdIha4jabcYcaSiabdMha5bGaayjkaiaawMcaaiabgIGiolqbfM6axzaafaaaaaaaaa@8DD7@

where Ω denotes the region within the t-tubule network, Ω' denotes the t-tubule membrane, Γ denotes the boundary between the t-tubule network and the interstitial space, *c*_0 _= 4 mM is the [K^+^] in the interstitial space, n^
 MathType@MTEF@5@5@+=feaafiart1ev1aaatCvAUfKttLearuWrP9MDH5MBPbIqV92AaeXatLxBI9gBaebbnrfifHhDYfgasaacH8akY=wiFfYdH8Gipec8Eeeu0xXdbba9frFj0=OqFfea0dXdd9vqai=hGuQ8kuc9pgc9s8qqaq=dirpe0xb9q8qiLsFr0=vr0=vr0dc8meaabaqaciaacaGaaeqabaqabeGadaaakeaaieqacuWFUbGBgaqcaaaa@2E27@ is the outward normal at the t-tubule membrane, *B *= 0.002 μm^-1 ^represents the net rate of K^+ ^release across the t-tubule membrane, *A *= 60 mM represents the reduction in K^+ ^release from voltage-gated K^+ ^channels due to t-tubule K^+ ^accumulation and *D *is the K^+ ^diffusion coefficient in free solution. The parameters *A *and *B *were chosen to reproduce qualitatively the macroscopic t-tubule [K^+^] gradient generated in the model by Wallinga et al. [4]. Eq. (11) was solved using the Matlab software package (The MathWorks) with the method of lines [30].

**Figure 5:**
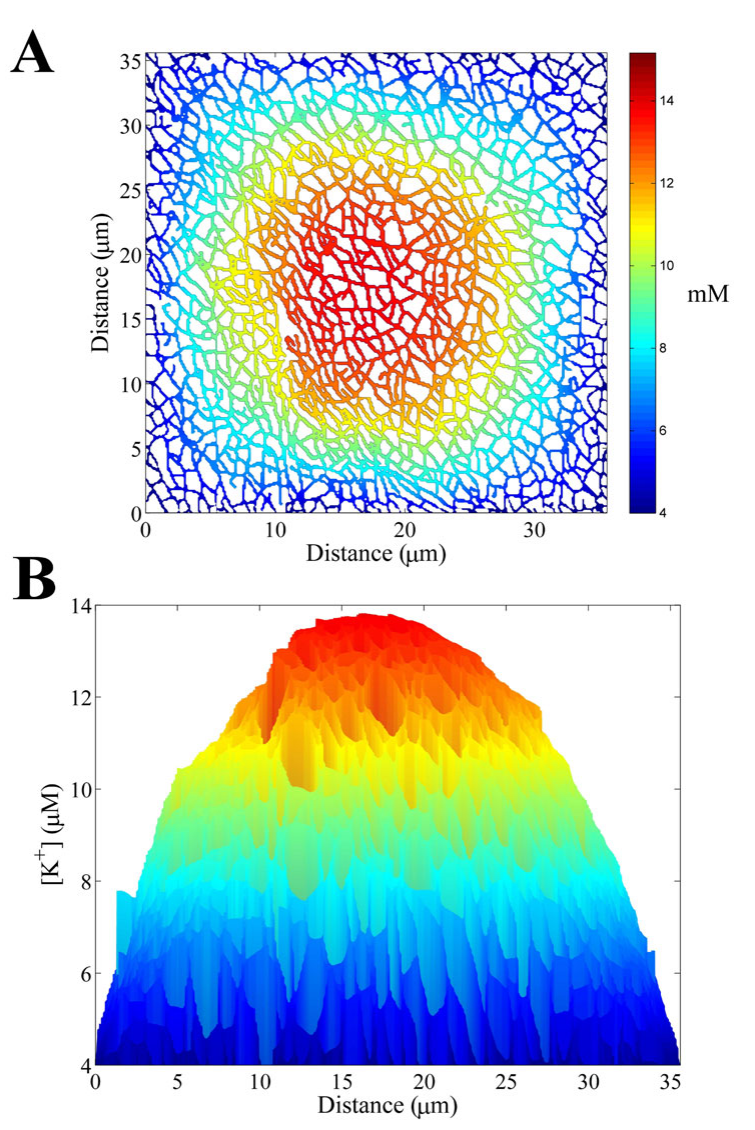
**A **The simulated steady state K^+ ^distribution within a 35 μm × 35 μm section of the frog t-tubule network during repeated high-frequency activation of skeletal muscle. **B **The K^+ ^profile across the fibre.

Skeletal muscle fibres have cross-sections that are polygonal with 3–7 sides [31]. For demonstration purposes we consider a fibre with a square cross-section. The simulated steady state [K^+^] distribution on a 35 μm × 35 μm square section of the frog t-tubule network is shown in Fig. 5. There is a significant [K^+^] gradient across the fibre and the [K^+^] at the centre of the network is approximately 14 mM. This is consistent with the computer modelling by Wallinga et al. [4], who predicted that the [K^+^] at the centre of a 40 μm diameter network increases to 12.5 mM after stimulation at 40 Hz for 1000 ms. Because the network is anisotropic and inhomogeneous, the [K^+^] distribution within the network is not symmetric. However, the level of asymmetry is small for this sized network and a homogenized description of the 35 μm × 35 μm t-tubule network can be safely employed to model the t-tubule system [4,32]. A large t-tubule network can therefore be considered as an isotropic irregular network.

**Figure 6:**
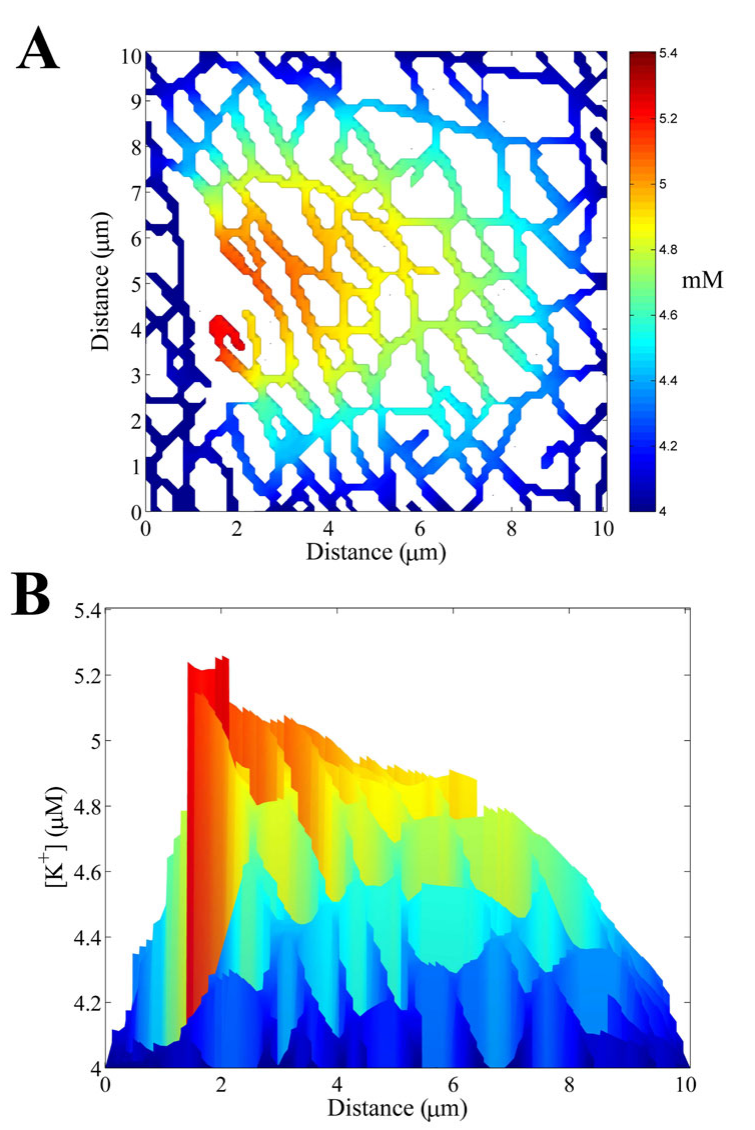
**A **The simulated steady state K^+ ^distribution within a 10 μm × 10 μm section of the frog t-tubule network during repeated high-frequency activation of skeletal muscle. **B **The K^+ ^profile across the fibre.

However, smaller t-tubule networks are expected to be more affected by network anisotropy and inhomogeneity. To investigate this we calculated the steady state [K^+^] distribution on the 10 μm × 10 μm square section of the frog t-tubule network shown in Fig. 6. The [K^+^] within the interior of this network is less than that for the larger fibre in Fig. 5 and smaller skeletal muscle fibres are therefore more resistant to membrane depolarization. The level of irregularity in the [K^+^] distribution within the network is also greater for this smaller network. T-tubule network anisotropy and inhomogeneity therefore potentially has a significant effect on the [K^+^] distribution in small muscle fibres.

## 3. Discussion

Experiments show that the skeletal muscle t-tubule network significantly impedes the diffusive transport of material through the t-tubules. Tortuosity measurements range from *τ *= 0.11 for Ca^2+ ^to *τ *= 0.21 for K^+ ^and Na^+ ^[5,6,21], which are significantly lower than the tortuosity factors calculated on theoretical grounds (0.32 ≤ *τ *≤ 0.5). Our work demonstrated why previous theoretical studies overestimate the t-tubule tortuosity factor. We calculated total tortuosity factors of *τ *= 0.19 and *τ *= 0.27 respectively for K^+ ^transport in human [8] and frog [7] skeletal muscle t-tubule geometries. Our theoretical estimate of the tortuosity factor, which includes geometric, hydrodynamic and non-planar effects, is consistent with experimental observations. A contributing reason for the difference in the tortuosity factor *τ *between the human and frog networks is that the latter network is a traced taut reconstruction of the network (i.e. the t-tubule branches do not contain wiggles). However, it is evident from scanned transverse sections in Fig. 2 that the t-tubule branches do contain wiggles and are therefore not taut. These t-tubule network wiggles were predicted to exist by Almers [5] based on the observation that muscle fibres can significantly shorten without damage (if muscle contracts under constant volume the t-tubule network must stretch). The network parameters obtained for the human network are therefore potentially more representative of skeletal muscle tissue.

We also observed that diffusion in the t-tubules is anomalous over short distances (i.e. does not obey Brownian motion) as a result of obstructed diffusion. The level of anomalous diffusion is described by an anomalous exponent (*α *= 1 represents Brownian diffusion) and we found anomalous exponents of *α *= 0.86 and *α *= 0.91 for the human and frog networks respectively. Obstructed diffusion is a localised phenomenon for an anomalous exponent (*α*) greater than the percolation threshold 0.697 [27]. For this reason the anomalous exponent *α *is also a function of the local t-tubule network neighbourhood defined by R*. The anomalous exponents reported in this paper are averages over the network geometry and for the frog network we found that 0.88 ≤ *α *≤ 0.95 and 5 ≤ R* ≤11 μm, which is a measure of network inhomogeneity. For small muscle fibres (diameter less than 10–20 μm) diffusion on the t-tubule network can be described by the fractional diffusion equation (Eq. 7), whereas for large muscle fibres (diameter greater than 20 μm) the homogenised diffusion equation (Eq. 1) provides an accurate approximation to diffusion on the t-tubule network.

A significant [K^+^] gradient is believed to be generated within the t-tubule network of skeletal muscle fibres during repeated high-frequency activation [3]. We found that the [K^+^] within the interior of the t-tubule network during high-frequency activation is greater for fibres with a larger diameter and therefore smaller skeletal muscle fibres are more resistant to membrane depolarization. We also found that fast-twitch muscle fibres have larger t-tubule tortuosity factors than slow-twitch fibres and are therefore better able to remove K^+ ^from the t-tubules via diffusive transport. Fast-twitch fibres are more susceptible to membrane depolarization and faster K^+ ^diffusion in the t-tubules therefore allows fast-twitch fibres to better control membrane depolarization. Because the t-tubule network is anisotropic and inhomogeneous, we also observed that the [K^+^] distribution generated within the network was irregular. Although the level of irregularity in the [K^+^] distribution within the network was small for larger sized networks (> 20 μm), it was significant for fibres of small diameter (< 20 μm). T-tubule networks within large muscle fibres can therefore be considered as isotropic irregular networks and a homogenized description of the network can be safely employed to model the t-tubule system [4,32].
